# The Student Grand Round: A Peer Teaching Initiative

**DOI:** 10.7759/cureus.60976

**Published:** 2024-05-24

**Authors:** Ahmed Nazari, Mariya Rajesh, Ibrahim Antoun, Mohamed Sheeraz Mohamed Azhar, Muhammad Hayat

**Affiliations:** 1 Internal Medicine, Northampton General Hospital NHS Trust, Northampton, GBR; 2 Internal Medicine, Northampton General Hospital, Northampton, GBR; 3 Cardiology, Royal Derby Hospital, Derby, GBR; 4 Internal Medicine, Southampton General Hospital NHS Foundation Trust, Southampton, GBR

**Keywords:** medical education (med ed) learning classroom integrated, peer-assisted learning, active teaching-learning, teaching service, safe patient handover, oral presentation skill, student education

## Abstract

Introduction

Oral presentation and public speaking skills are poorly emphasised in the medical school curriculum. The student grand round was created to tackle this deficiency by changing the way in which students are taught, from traditional lecture-based learning to interactive small-group peer-to-peer teaching. This approach encourages students to become responsible for their own learning, develop their public speaking and teaching skills, as well as identify and address gaps in their knowledge.

Aims

The primary aims of this study were to determine the understanding of students before and after peer teaching, including retention of concepts via quiz scores and confidence of students in giving SBAR (Situation, Background, Assessment, Recommendation) handovers. The secondary aim is to determine the place of student-led grand round teaching in the medical curriculum as a means of developing teaching skills and encouraging active learning.

Methods

A cohort of 21 third-year medical students from Leicester University attended a weekly peer teaching programme where students presented a case they had encountered during their clinical attachment. Peer teachers were required to research some background and pathophysiology regarding the topic and teach in an interactive manner and create discussion regarding the topic. The students then summarised the case and practised the skill of concise handovers using the SBAR format. Knowledge and understanding were assessed with an interactive quiz, and feedback via a survey was gathered before and after sessions. Each student engaged in case discussion and received input from a specialty registrar regarding their presentation skills, case knowledge, and SBAR handover.

Results

Individual and combined session analysis demonstrated a significant improvement in scores across understanding the topic and confidence in SBAR. Student recommendation for the session cumulatively was significant (p=0.02); however, comparison of medical student recommendations of individual sessions did not yield statistically significant results. There was a significant improvement in the overall quiz score (p=0.045), and average scores improved from 51% to 70% (p=0.043). There was a significant increase in the mean quiz result after the first two sessions (28-55% (p=0.002) and 56-85% (p=0.0001), respectively).

Summary

The student grand round is a promising teaching initiative that capitalises on peer teaching, a valuable learning theory that centres around students taking on the role of teachers to instruct their peers. Results from this study have shown that this method of collaborative teaching is effective in improving the understanding of medical topics, increases confidence in public speaking and precise handover skills, and therefore better prepares medical students for their career as future clinicians.

## Introduction

Oral presentation and public speaking skills are poorly emphasised in the medical school curriculum despite the General Medical Council stating that medical students must demonstrate basic teaching skills as part of their professional development [1]. One way to tackle this deficiency is by changing the way in which students are taught, from traditional lecture-based learning to interactive small-group teaching. One such method is peer teaching, a form of active learning that centres around students taking on the role of teachers to instruct their peers. Peer teaching is proven to improve conceptual understanding and enables the development of problem-solving and presentation skills [1,2], all of which are crucial to doctors. Cognitive congruence theory states that students learn more effectively from peer teachers as they are more likely to comprehend the student's current knowledge, identify areas of difficulty, and formulate creative solutions to learning [3]. Peer tutors can foster a relaxed communal learning environment where students can freely discuss and share ideas while also incorporating new experiences and approaches to the curriculum [4]. Tutors are also able to reinforce their knowledge of the topic being taught while also developing their own teaching skills and receiving constructive feedback [5].

Case-based discussion and problem-based learning are forms of peer teaching currently practised widely in the medical curriculum [6]. This approach encourages students to become responsible for their own learning, an essential skill for higher training in the medical field. Interest is generated by focusing on "real-life scenarios" and encourages a constructional and deep approach to learning whereby students identify and build on gaps in their knowledge [6]. To address this, the student grand round was developed to create an environment for students where they take control of their learning and understanding. This project involved third-year medical students from Leicester University who, in pairs or groups of three, would create a short teaching presentation regarding a case they had been involved with during their clinical attachment. The students were required to research and learn the background and pathophysiology of the case and present and teach this to their peers; they then summarised the case and practised the skill of succinct handovers using the SBAR (Situation, Background, Assessment, Recommendation) format. Each presentation was supervised by a specialty registrar who discussed the case allowing for additional learning opportunities and provided feedback regarding the students' teaching and presentation skills. The students' knowledge and understanding were tested with an interactive multiple-choice quiz before and after each session. 

Aims

The primary aim of this study is to determine the understanding that students had of medical topics taught in the sessions before peer teaching and observe, using multiple-choice quiz scores pre- and post-session, whether peer teaching increases retention and understanding of these topics. The secondary aim of the study is to allow students to practise presenting SBAR handovers and increase confidence in public speaking. Finally, the tertiary aim is to determine the place of student-led grand round teaching in the medical curriculum as a means of developing teaching skills and encouraging active learning. 

## Materials and methods

An initial online survey was conducted in a questionnaire format, to assess medical students' interest and knowledge of peer teaching. There were 75 responses from third- and fourth-year medical students from Leicester University and Oxford University with a 98% interest to partake in the initial peer teaching quality improvement study. A cohort of 21 third-year medical students from Leicester University attended a weekly peer teaching programme. Students were required to formulate a case presentation by researching and learning the background and pathophysiology of the case and present this in an interactive way to teach their peers and create discussion regarding the topic. Using feedback from an online questionnaire, improvements were made in subsequent sessions such as the addition of an interactive quiz and senior feedback. 

Students covered conditions from urology, general surgery, vascular surgery, orthopaedic surgery, cardiology, respiratory medicine, gastroenterology, renal medicine, rheumatology, endocrinology, elderly care medicine, and anaesthetic. Various topics from various medical and surgical specialties were given over the study period. 

The students were then tasked with summarising the case and practised the skill of concise handovers using the SBAR format. Their knowledge and understanding of recipients of the case presentations were assessed with an interactive multiple-choice quiz with questions formulated from the students' medical curriculum before and after each session, and feedback via an online survey was gathered before and after sessions. Each student engaged in case discussion and received input from a specialty registrar regarding their presentation skills, case knowledge, and SBAR handover. 

This study assessed the student's understanding of the topics before and after the sessions using online interactive quizzes, confidence in presenting, and SBAR handover pre- and post-sessions.

Statistical analysis

Continuous variables are expressed as mean and standard error of the mean (SEM). Categorical variables are expressed as numbers and percentages (%). Pearson's χ2 or Fisher's exact test was used for categorical variables between groups. Student's t-test and the Kruskal-Wallis test were used to compare continuous variables between the groups depending on the normality of the distribution. Paired t-tests or Wilcoxon signed-rank tests were used to compare the improvement in students' understanding before and after the sessions. A one-way analysis of variance (ANOVA) test was conducted to assess the difference between scores from different sessions with post hoc Bonferroni (for parametric data) or Dunn's test (for non-parametric data) conducted if ANOVA demonstrated significant results. Statistical analysis was performed using GraphPad Prism V9.5 for Mac (San Diego, California, USA; www.graphpad.com). 

## Results

The four sessions involved 84 attendees (25-21-20-18 students, respectively, in the order of the session). All students answered feedback questions. Table 1 demonstrates a detailed analysis of the mean scores given by the students before and after the sessions. Individual and combined session analysis demonstrated a significant improvement in scores across understanding the topic and confidence in SBAR across all sessions individually and collectively, as demonstrated in Table 1. When individual sessions were compared, no session had a superior improvement in topic understanding (F(DFn, DFd)=1.6 (3, 81); p=0.18) or confidence in SBAR (F(DFn, DFd)=3.9 (3, 81); p=0.76). The quiz feedback scores were statistically similar between individual sessions at the beginning (F(DFn, DFd)=0.05 (2, 57); p=0.95) and at the end (F(DFn, DFd)=1.22 (2, 57); p=0.3). While comparing the proportion of students recommending the session was significant when all sessions were compared (p=0.02), comparing individual sessions did not yield a statistical difference as demonstrated in Table 2. 

**Table 1:** Detailed analysis of all three sessions with the mean scores given by the medical students. Each area was scored from 1 to 5, 1 meaning poor and 5 meaning excellent *Represents values which were not assessed in all sessions and the combined sessions column was assessed with the remaining data. SBAR: Situation, Background, Assessment, Recommendation

	Session 1 (n=25)	Session 2 (n=21)	Session 3 (n=20)	Session 4 (n=18)	All sessions
Topic understanding pre	3±0.15	2.9±0.17	2.7±0.17	2.8±0.2	2.8±0.08
Topic understanding post	4.4±0.1	4±0.13	4.1±0.12	4±0.1	4.1±0.06
P-value	<0.001	<0.001	<0.001	0.002	<0.001
SBAR confidence pre	2.5±0.19	3±0.18	3.3±0.14	3.4±2.3	2.8±0.09
SBAR confidence post	3.2±0.17	4±0.15	4±0.14	4.1±0.16	3.6±0.09
P-value	<0.001	<0.001	0.009	0.015	<0.001
Beginning quiz*		4.3±0.13	4.4±0.16	4.4±0.35	4.4±0.1
End quiz*		4.3±0.15	4.4±0.15	4.6±0.5	4.4±0.1
Quiz explanation*				4.6±0.3	4.6±0.3
Senior feedback*				4.3±0.4	4.3±0.4
Rate	4.2±0.17	4.3±0.13	4.1±0.13	4.2±0.4	4.2±0.1
Recommending the session	17 (68%)	19 (90%)	19 (95%)	18 (95%)	73 (85%)

**Table 2:** Individual comparison of the proportion of medical students recommending the teaching sessions *Multiple comparison was conducted using the Kruskal-Wallis test **Post hoc analysis using Dunn's test was conducted

Session comparison*	P-value**
1 vs. 2	0.18
1 vs. 3	0.06
1 vs. 4	0.07
2 vs. 3	0.99
2 vs. 4	0.99
3 vs. 4	0.99

Quiz results details are demonstrated in Table 3. Overall average scores improved from 51% to 70% (p=0.043). There was a significant increase in the mean quiz result after the first two sessions (28-55% (p=0.002) and 56-85% (p<0.0001), respectively). Session 3 and 4 mean quiz scores were not significantly increased after the session. The quiz score was 85% after session 2, which had the best score improvement of 29%.

**Table 3:** The average quiz scores of students' groups before and after the sessions. Quizzes were answered in groups

Session (participants)	Pre-average score (%)	Post-average score (%)	P-value
1 (n=25)	28	55	0.002
2 (n=21)	56	85	<0.0001
3 (n=20)	64	70	0.45
4 (n=19)	55	68	0.08
Overall (n=85)	51	70	0.043

## Discussion

This study showed that individual and combined session analysis significantly improved scores across the understanding of topics and confidence in SBAR handover skills across all sessions, individually and collectively. This can be explained through social theories of learning; the environment and the community in which students participate in are essential to motivate and encourage development through consolidating existing knowledge and acquiring new knowledge [7-9]. Peer teaching significantly improved student skill performance compared to expert and facilitated teaching and self-study as shown in a meta-analysis [10] and a systematic review on peer teaching [11]. 

Medical institutions that are dealing with an increasing student body but a static faculty size may find it appealing since it draws on the social and cognitive congruence between the student and instructor. Peer instructors can conduct problem-based learning sessions, deliver lectures on certain subjects, and assist individual peers [8]. Student interaction through peer teaching facilitates discussion through questioning and explaining existing concepts. Furthermore, exchanging ideas and collective problem-solving assists in the affirmation of knowledge.

One popular method of peer teaching is the "jigsaw" technique, in which each student specialises in a specific topic and subsequently collaborates with classmates to exchange their expertise. This technique fosters a deeper understanding of the subject matter and promotes the development of communication and teamwork skills [12]. Collaborative learning and the education reform for healthcare professionals demand that the emphasis be shifted from memorising facts to investigating, analysing, assimilating, and synthesising knowledge. Peer teaching can be used to engage students in clinical teaching effectively as shown by a meta-analysis of 44 randomised controlled trials on peer teaching which showed promising results; peer teaching had similar knowledge acquisition as compared to expert and facilitated teaching [8].

However, when individual sessions were compared, no session had a superior improvement in topic understanding; this can be explained as new topics were taught in each session. Students' recommendation for the session cumulatively was significant (p=0.02). Despite this, comparison of medical student recommendations of individual sessions did not yield statistically significant results, as shown in Table 2. This may be explained by changes implemented between the sessions. There was less senior input in the earlier sessions than in the last few sessions, as shown in Table 1. 

There was a significant improvement in the overall quiz score (p=0.045) when comparing pre- and post-session quiz scores as shown in Table 3. The literature has shown effective testing, active recall, and extending repetition intervals ensure efficacious long-term memory retention and reduced memory decay for information over time [13,14]. The student grand round is a multi-modular teaching project that benefits from combining various interactive teaching concepts and techniques to aid learning.

There were limitations to this study as there was no standardisation or set curriculum on what teaching skills should be required of medical students. A competency framework would give medical educators a shared set of objectives to focus their research efforts on, as the development of such abilities is a logical stage in students' educational growth in their academic lives and careers. A study on third-year medical students' peer teaching and mentoring experience highlighted the significance of establishing educational settings that cultivate peer instruction and mentoring [15]. Such experiences could contribute to ongoing development in a student's capacity and as a medical practitioner. Furthermore, we recognise that the initial sample size of 21 students is small; this study was completed as a proof of concept with the full study population involving 85 medical students. A post hoc sample size power analysis was completed and shown to have a confidence level of 95% that the real value is within ±10% of the measured/surveyed value of 97, which is slightly higher than our sample size and is a limitation to our study. 

Incorporating peer teaching into the classroom requires thoughtful planning and teacher guidance to ensure accurate information is shared. While it may not replace the role of the instructor, it has been shown to complement traditional teaching methods and create a dynamic and interactive learning environment that benefits all students involved [16]. In this project, the peer teaching session was scheduled into the students' timetable on a weekly basis as a confluence of knowledge gained during their medical placement. The student grand round is one format of peer teaching that can be easily adapted into the medical curricula and scheduled into students' teaching as an adjunct to traditional teaching methods. 

Collaborative learning through peer teaching promotes a communal learning atmosphere where students actively contribute to each other's educational needs, fostering a shift from teacher-centred to student-centred learning. As students take on teaching roles, they break down complex concepts, deepening their understanding and boosting retention. This approach heightens engagement, motivating students to study thoroughly. Communication skills improve as students articulate ideas for peers, adapting explanations effectively. Diverse perspectives from classmates enrich the learning experience while simultaneously reducing anxiety in a relaxed environment, which can encourage open questioning. This was demonstrated in the quasi-experimental study which found that peer teaching significantly reduced learning anxiety among nursing students [17]. Equipping students with fundamental teaching skills is essential to their careers as physicians. 

## Conclusions

The student grand round is a promising teaching initiative that capitalises on peer teaching, a valuable learning theory that centres around students taking on the role of teachers to instruct their peers. The student grand round is particularly relevant in medical education due to the complex nature of medical concepts and the need for effective communication among future healthcare professionals. Medical students often collaborate to understand intricate medical topics, practise clinical skills, and prepare for exams, and this approach allows students to share their knowledge, clarify doubts, and learn from diverse perspectives. Further research into the longevity and resilience of any new teaching initiative is required to ensure its sustainability. Results from this study have shown that this method of collaborative teaching is effective in improving the understanding of medical topics, increases confidence in public speaking and precise handover skills, and therefore better prepares medical students for their careers as future clinicians. 
